# Are OMERACT recommendations followed in clinical trials on fibromyalgia? A systematic review of patient-reported outcomes and their measures

**DOI:** 10.1007/s11136-022-03261-5

**Published:** 2022-10-01

**Authors:** Annika Döhmen, Milan Kock, Felix Fischer, Matthias Rose, Alexander Obbarius, Christoph Paul Klapproth

**Affiliations:** 1 Department of Psychosomatic Medicine, Center for Internal Medicine and Dermatology, Charité – Universitätsmedizin Berlin, Charitéplatz 1, 10117 Berlin, Germany; 2 Department of Quantitative Health Sciences, Medical School, University of Massachusetts, Worcester, MA USA; 3 Dornsife Center for Self-Report Science, University of Southern California, Los Angeles, USA

**Keywords:** Fibromyalgia, Health related quality of life, Patient-reported outcomes, Rheumatology

## Abstract

**Purpose:**

Patient-Reported Outcomes (PROs) and its measures (PROMs) are key to outcome assessment in Fibromyalgia (FM) trials. The aim of this review was to investigate which domains and instruments were assessed in recent FM trials and to compare them to recommendations by the Outcome Measures in Rheumatology (OMERACT) initiative. In addition, we investigated the overlap with a generic health assessment approach, i.e. eight domains suggested by the Patient-Reported Outcome Measurement Information System® (PROMIS®).

**Methods:**

In compliance with the Preferred Reporting Items for Systematic Reviews and Meta-Analyses (PRISMA) guidelines, a systematic literature search in scientific databases including PubMed, PsycInfo, and Embase was conducted to identify studies that assessed at least two dimensions of health-related quality of life (HRQoL) from 2015 to June 2022. Non-randomized and randomized controlled trials were included in the analysis. We extracted PROs and PROMs used in each study.

**Results:**

From 1845 identified records, 107 records out of 105 studies met the inclusion criteria. Studies investigated 50 PROs using 126 different PROMs. Most frequently assessed domains were pain, depression, fatigue, and anxiety (> 95% of the studies). The disease-specific FIQ was the most frequently applied PROM (82%). Overall, only 9% of the studies covered all domains deemed mandatory by OMERACT. Very few studies covered all eight generic health domains suggested by PROMIS.

**Conclusion:**

The majority of trials covered most OMERACT domains or generic PROMIS health domains. There was, however, great variability in the instruments used to assess the domains, which points at a limited degree of standardization in the field.

**Supplementary Information:**

The online version contains supplementary material available at 10.1007/s11136-022-03261-5.

## Plain English summary

Fibromyalgia is a chronic pain condition characterized by pain in various areas of the body and other symptoms such as fatigue or concentration problems. There are no established laboratory values or examination techniques for detecting or monitoring the disease, which is why patient-reported outcomes (PROs) are particularly important. A PRO is a health outcome, such as the severity of depression or pain, self-reported by the patient who experienced it. The Outcome Measures in Rheumatology (OMERACT) initiative recommended assessing PROs in clinical studies on fibromyalgia to determine whether a treatment was successful. The Patient-Reported Outcome Measurement Information System® (PROMIS®) initiative provides “generic” measures of PROs (PRO measures, PROMs) that can be used in many conditions including the rheumatologic ones. The aim of this study was to investigate which PROs were assessed in clinical trials on fibromyalgia and which PROMs were used to assess each PRO. In addition, we aimed to find out how current practice of PRO assessment corresponds to the recommendations by OMERACT and PROMIS.

To achieve this goal, we looked at 107 different publications from 105 different studies on fibromyalgia patients. We found that the majority of PROs recommended by OMERACT were captured in these studies, but only few studies met the recommendations in full. Most of the studies included PROs that reflect the general PROMIS health domains. Noteworthy, many different PROMs were used for assessing the same PROs. These results suggest that while there is consensus on the use of PROs, there is still a long way to go towards standardizing the instruments used.

## Introduction

Fibromyalgia (FM) is a chronic health condition that severely affects various areas of health-related quality of life (HRQoL) including physical, social, and emotional well-being. Due to the wide variety of clinical phenotypes and due to the unavailability of biomedical markers, treatment monitoring poses a significant challenge. Therefore, recommendations emphasize assessing patient-reported outcomes (PROs) as the most informative and efficient way of determining, whether a patient's health has deteriorated, remained the same, or improved over time during treatment. Unlike for many other chronic health conditions[1] there is to date no consensus on a specific set of PRO measures (PROMs) for fibromyalgia that should be used in research or clinical settings, which complicates the comparability across clinical settings and studies[2]. Limited comparability of outcome measures, for example, renders pooling of data in meta-analyses inconvenient and increases the risk of bias [3, 4]. To capture the current practice of outcome assessment in clinical trials, in the present study we aim to collect information on domains and instruments that have been used in previous studies.

In an effort of standardization, the Outcome Measures in Rheumatology (OMERACT) initiative has suggested various core sets of outcome measures that allow for better comparison across clinical trial results in Rheumatology. The outcome set for FM was introduced in 2009 [5] and includes groups of domains that should be assessed with graded priority: pain, tenderness, fatigue, patient global health, multidimensional function, and sleep disturbance were regarded as mandatory for all clinical trials, whereas depression and cognitive dysfunction may be assessed in some FM trials. Stiffness and anxiety, as well as the non-PRO markers cerebrospinal fluid biomarkers and functional imaging were regarded as optional for research purposes [6]. In accordance with the general OMERACT approach, the core set does not include recommendations for one specific instrument per domain. Instead, the OMERACT working group suggested a variety of instruments per domain that meet predefined quality criteria [6]. Many instruments have been designed for disease-specific application. That is, the questions are specific for FM and these instruments cannot be used in other (rheumatological) conditions.

The most widely used disease-specific instrument for FM is the Fibromyalgia Impact Questionnaire (FIQ), which covers many of the domains that are relevant for FM patients. The first version was introduced in 1991 and several revised versions have been developed since then [7]. This version covers a broad range of domains including physical functioning, work status, depression, anxiety, sleep, pain, stiffness, fatigue, and well-being as described by the author [7]. In 1997 and 2002, modifications regarding the scoring were implemented [8]. The revised FIQ (FIQ-R), published in 2009, additionally covers the domains memory, tenderness, balance, and environmental sensitivity [9]. The FIQ and its revised versions have been translated into several languages and it has been used in trials around the globe.

In recent years, a more generic approach to PRO measurement has been increasingly favoured, where disease-independent instruments are preferred over disease-specific ones. The U.S. National Institutes of Health funded Patient-Reported Outcome Measurement Information System® (PROMIS®), for example, has introduced a framework of health domains with the aim of standardizing health outcomes [10]. PROMIS core domains include physical, emotional, and social health aspects, which are relevant for almost every health condition including rheumatologic diseases such as FM. PROMIS has invested great efforts in developing psychometrically sound and efficient PROMs for each domain which is why it has been increasingly used in many clinical and research settings in recent years. A great advantage of the PROMIS approach is that conventional “legacy” instruments can be linked to the PROMIS metric, which facilitates comparability across different measurement systems [11, 12]. The metric used by PROMIS is the *T*-Score metric where 50 reflects the population average with a standard deviation of 10 [13].

In the present review, we investigate which PROs were selected as treatment outcomes and which PROMs were used to assess these outcomes in clinical trials of FM patients since 2015. We compare the selection of domains and instruments in these studies to the OMERACT recommendations [5]. Furthermore, we investigate whether the assessed domains were covered by a generic health assessment (i.e. PROMIS Profile). The PROMIS 29 profile covers eight health domains: anxiety, depression, pain intensity, pain interference, physical function, ability to participate in social roles and activities, sleep disturbance, and fatigue [10, 14]. These primary objectives were augmented by some more specific questions that were deemed relevant for PRO assessment and reporting of FM studies. Guidelines of the American Psychological Associations recommend including key psychometric information for each instrument in publications [15, 16]. Thus, we examined whether validity and reliability data on instruments provided in the articles are included in this systematic review. Moreover, motivated by the frequent use of the FIQ in FM trials, we investigate the use and interpretation of different versions of the FIQ in detail. Finally, we evaluate whether the studies included instruments that would allow determining the health state utilities (HSU) of the intervention under investigation. Data from clinical trials are increasingly used for evaluating the health utility of new treatments in secondary analyses. Thus, assessment of instruments that can be used for these analyses is deemed important. Preference-based PROs, such as the EQ-5D and the SF-6D, can not only be used to describe HRQoL but also to measure HSU for quality-adjusted life years (QALY) in the economic evaluations of treatments[17].

The overarching goal of this systematic review is to provide a substantial overview on the current practice of PRO assessment in clinical trials in FM patients in order to indicate a potential need for further standardization of outcome assessment in this population. Advancing standardization is essential to ensure better comparability of treatment outcomes across studies and settings.

## Methods

We report our results in accordance with the Preferred Reporting Items for Systematic Reviews and Meta-Analyses (PRISMA) guidelines as well as with the Cochrane Recommendation for Systematic Reviews [18]. Graphics were created using Microsoft Word 2019 and Microsoft Power Point 2019.

### Eligibility criteria

Eligibility criteria were defined by the research team (AD, MK, AO, FF, CPK). Studies were included if they were written in English or German and met the following criteria:

- Study population needed to consist of adults (i.e. age of participants ≥ 18 years).
- FM had to be the defining condition of the study population.
- Randomized controlled clinical trials (RCTs) and controlled clinical trials, in which the allocation of the participants was not randomized (CTs) were eligible for inclusion [19].
- Publication needed to be between 2015 and June 2022. The search span was limited to ensure that the OMERACT recommendations, which were published in 2009, would be broadly known among researchers in this field [5].
- Studies had to assess at least two HRQoL domains according to PROMIS.
- Patient-reported outcomes had to be measured by self-reports.

Studies were excluded from this systematic review if they met the following exclusion criteria:

- Studies that did not assess HRQoL by self-reports (i.e. clinician-reported, based on interview).
- Studies that evaluated FM as a comorbidity or investigated multiple other health conditions in addition to FM.
- Clinical trials without a control condition, as well as editorials, commentaries, conference abstracts, reviews, and unpublished studies.
- Studies that were defined as secondary analyses of previously published articles outside of the literature research period.

### Information sources

A systematic literature research was conducted on 7th December 2020 and updated on 25th June 2022 (AD and MK). Databases included Pubmed (Medline), PsycINFO, and Embase, using the Ovid interface.

### Search strategy

The search strategy followed the same strategy in all databases. The format of the search term was slightly adapted in accordance with the database requirements. The search term consisted of the term “fibromyalgia” in abstract and/or title in combination with “quality of life”, “health-related quality of life”, “health utility”, “health utilities”, “health state utility”, “health state utilities”, or “preference-based” in text word search, including the title, abstract, MeSH terms, MeSH subheadings, substance names, and other terms apart from the full text of the article. Filters were used to present only articles in English or German language and to limit the results to articles that were published since 2015.

### Study selection process

Titles of the records were examined for duplicates using the reference management software EndNote X9 [20]. Titles of remaining records were then screened by two reviewers separately (AD and MK). If titles revealed that articles did not meet the defined criteria, studies were excluded. Next, abstracts were divided into two portions and each half was screened by one reviewer. Studies that did not meet the defined criteria were excluded. During the screening process, AD and MK reached a consensus decision on whether to include or exclude a study. If there was disagreement between the two researchers, the study was discussed within the research team (AD, MK, AO, CPK) and a decision was reached together. Observational studies and clinical trials without a control group were identified and excluded. Full text versions of all articles were then obtained and screened. Again, articles that did not match the inclusion criteria were excluded and unclear cases were discussed within the study team as described above.

### Data collection process

Data to be extracted were determined based on Cochrane recommendations [21]. Data were extracted according to predefined criteria by AD and MK independently and collected in a Microsoft Excel file to ensure standardized data collection.

Extracted data included:

- Information on general study characteristics (type of study, country, year of publication)
- Data on study participants (number of participants, diagnostic criteria for FM, age, sex)
- Patient-Reported Outcomes (PROs) used in the study
- PRO measures (PROMs) used in the study (including disease-specific/generic, version)
- Whether studies reported psychometric criteria for the instruments collected. Psychometric characteristics included several aspects of reliability (internal consistency, test–retest reliability), validity (construct validity including convergent validity and discriminant validity, predictive validity), and sensitivity to change/responsiveness. To facilitate readability, we subsume responsiveness under the term sensitivity to change and follow the definition by the COSMIN group (“the ability of an instrument to detect change over time in the construct to be measured”) [22].

### Synthesis methods

We identified all PROMs that were used to measure study outcomes. In addition, we identified the constructs that were measured with these instruments. The PROQOLID database (https://eprovide.mapi-trust.org/) and original instrument development articles were used to obtain the information on which items and subscales represent which constructs. In rare cases, if one subscale included items from various HRQoL domains, single items were assigned to specific domains. To find out whether the studies have assessed the domains recommended by OMERACT (i.e. the recommended FM outcome set) and included in the PROMIS Profile, the domains covered by each study were compared to the OMERACT and PROMIS domains [5, 6, 23]. If domains did not correspond to either construct, the original domains of the PROs were added to the list of domains.

## Results

### Search strategy results

**Fig. 1:**
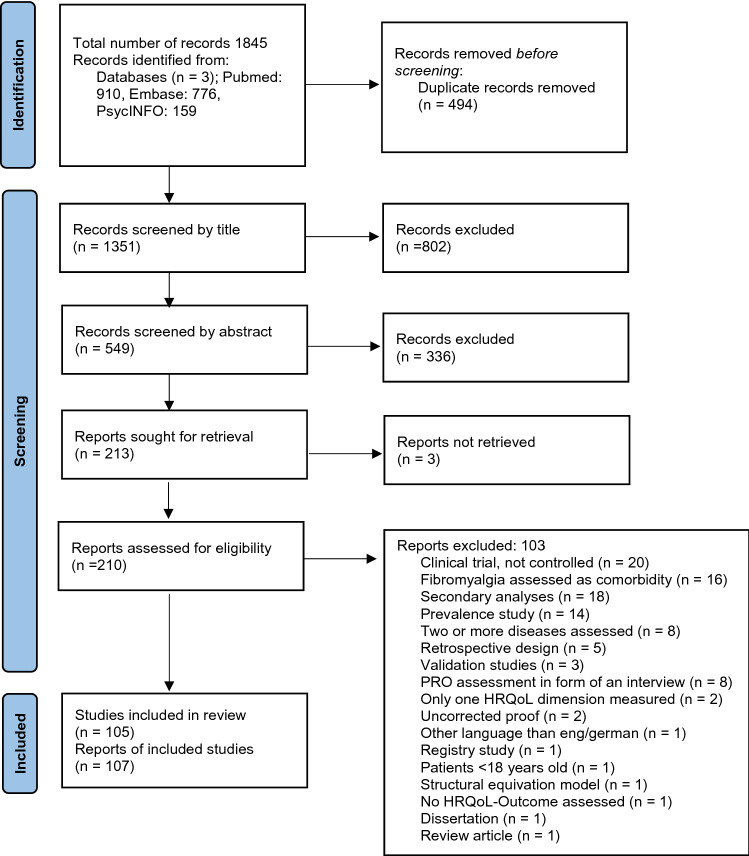
PRISMA flowchart: Overview of the study selection process *PRO* patient-reported outcome; *HRQoL* health-related quality of life

The initial database research in December 2020 returned 1391 individual citations. After screening of titles and abstracts and the consecutive full text review of 182 articles, 85 studies (63 RCTs and 22 CTs) were initially identified to be included in this systematic review. The search was updated in June 2022. A total number of 1845 articles were identified at first, of which 910 records were obtained from Pubmed (Medline), 776 records were obtained from Embase, and 159 records were obtained from PsycINFO. A number of 494 duplicates were removed. After screening the titles and abstracts, 213 articles remained. Three articles were not available as full text. Following the full text review of 210 articles, a total number of 107 articles were included in this systematic review (Fig. 1). These articles reported on 105 different studies (83 RCTs and 22 CTs)[24–130].

### Study characteristics

The average sample size of all studies was 85.0 (*S.D*. = 90.0). The sample sizes of the RCTs (*M* = 87.4, *S.D.* = 92.4) were somewhat larger that the sample sizes of the CTs (*M* = 75.6, *S.D.* = 79.7). The proportion of women included in the analysed studies ranged from 88.6 to 100%. Further study characteristics are provided in the online supplemental material.

### Domains

Overall, the 105 included studies covered 50 domains. After allocating the PRO domains to the OMERACT core domains, 40 domains remained that were not included in the OMERACT recommendations [5].

The OMERACT domains most frequently assessed were pain (98.1%), depression (98.1%), fatigue (96.2%), and anxiety (95.2%). At least one subdomain of multidimensional function, including physical and social functioning, was collected in 96.2% of the studies. Of the OMERACT core domains, tenderness was least frequently assessed (17.1%). Of the domains deemed optional by OMERACT (i.e. depression and cognitive function), depression was captured in 98.1% and cognitive function was assessed in 28.6% of the studies. The PRO domains regarded as optional research domains by OMERACT were assessed in the majority of studies (stiffness 82.0%, anxiety 95.2%).

In summary, only 8.6% (9 studies) of the studies covered all mandatory domains as recommended by OMERACT [26, 31, 41, 42, 75, 76, 87, 112, 130] and only 6.7% (7 studies) covered all mandatory and optional domains [26, 31, 41, 42, 76, 112, 130]. Remarkably, although the OMERACT recommendations have been available since 2009 [5], only eight studies referred to those recommendations in the methods section or in the discussion [30, 56, 73, 79, 80, 113, 125, 126]. Seven studies cited the OMERACT core set because they explicitly conformed to their recommendations [30, 56, 73, 80, 113, 125, 126].

**Fig. 2:**
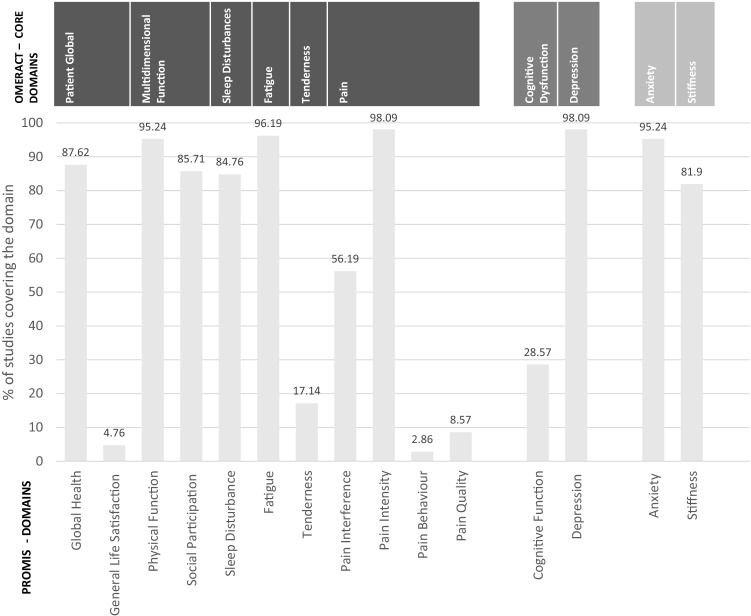
Coverage of OMERACT and PROMIS domains by included studies X-axis (top): OMERACT core domains, divided into inner (dark grey), outer (lighter grey) and outermost circle (lightest grey). X-axis: (bottom) PROMIS domains. Y-axis: Domain coverage in % *OMERACT *outcome measures in rheumatology; *PROMIS* patient-reported outcomes measurement information system

The allocation of PROMIS domains to the OMERACT core set is shown in Fig. 2. The PROMIS Profile domains cover all OMERACT fibromyalgia domains [131], except for tenderness and stiffness. Some of the assessed PRO domains such as self-efficacy (in 4.8% of the studies), positive affect (1.9%), and sexual function and satisfaction (1.9%) are included in the PROMIS framework but are not part of the OMERACT core domains. 27 domains could neither be matched to OMERACT nor to PROMIS, however, only eight of these additional domains have been assessed more than once. Of note, the domain sensitization to light and noises, although not part of the OMERACT recommendation, has been assessed in 15.2% of the included studies because this domain is part of the FIQ-R [5].

### PRO measures

A total of 126 different PROMs were used to assess 50 domains. On average, 5.06 (*S.D.* = 2.89) PROMs were used per study. Considering the use of different versions of these questionnaires, 140 different instruments were collected. For example, the FIQ was frequently administered in its versions from 1990 to 2009 [9]. Even though the majority of the PROMs were generic, the most frequently collected instrument was the disease-specific FIQ, which was used in 81.9% of the studies. Followed by the generic Short Form Health Survey (SF-36), which was assessed in 44.8% of the studies. Only ten studies did apply neither the FIQ nor the SF-36 [32, 69, 73, 82, 83, 98, 99, 105, 109, 128], although Pazzi et al. and Macian et al. [83, 105] did use the 12-item Short Form of the SF-36 (i.e. SF-12). Besides these multidimensional instruments, a Visual Analogue Scale (VAS) for Pain was administered in 51.4% of the studies. A VAS was also used in five studies to assess fatigue and in two studies to assess sleep. Noteworthy, 78 PROMs were only used in one study each. The frequency of instruments used in two or more studies is shown in Table 1.

**Table 1:** Frequency of the assessment of PROMs used in at least two studies

Questionnaire	Frequency of assessment
Fibromyalgia Impact Questionnaire (FIQ)	86
FIQ 1991	38
Revised FIQ (FIQ-R) 2009	16
FIQ version not specified	32
Visual Analogue Scale (VAS) Pain	54
Short Form Health Survey 36 (SF-36)	47
SF-36 version 2	1
SF-36 version not specified	45
SF-36 subscale fatigue	1
Beck’s Depression Inventory (BDI)	22
BDI	18
BDI-II	4
Hospital Anxiety and Depression Scale (HADS)	21
Pittsburgh Sleep Quality Index (PSQI)	21
PSQI-short form	3
PSQI-long form	18
Widespread Pain Index (WPI)	17
Pain Catastrophizing Scale (PCS)	16
FM Symptom Severity Scale	15
Brief Pain Inventory (BPI)	9
BPI long form	6
BPI short form	3
Fatigue Severity Scale (FSS)	9
Numeric Rating Scale Pain (NRS Pain)	9
Patient Global Impression of Change (PGIC)	9
European Quality of Life 5 Dimensions (EQ-5D)	8
EQ-5D-3 Levels	2
EQ-5D-5 Levels	4
VAS-Scale of EQ-5D	2
Health Assessment Questionnaire (HAQ)	7
HAQ-9	1
HAQ-Disability Index (HAQ-DI)	1
HAQ-Improved (HAQ-I)	1
HAQ (version not specified)	4
Short Form Health Survey 12 (SF-12)	6
SF-12 version 2	1
SF-12 version not specified	5
Strait Trait Anxiety Inventory (STAI)	6
Visual Analogue Scale (VAS) Fatigue	5
Beck’s Anxiety Inventory (BAI)	4
Center for Epidemiological Studies Depression Scale (CFESDS)	4
Coping Strategies Questionnaire (CSQ)	4
CSQ subscale “catastrophizing”	1
McGill Pain Questionnaire (MPQ)	4
MPQ long form	2
MPQ short form	2
Perceived Stress Scale (PSS)	3
PSS	1
PSS-10	2
Satisfaction with Life Scale (SWLS)	4
Epworth Sleepiness Scale	3
Fear Avoidance Beliefs Questionnaire (FABQ)	3
Five-Facets of Mindfulness Questionnaire (FFMQ)	3
Insomnia Severity Index (ISI)	3
Multidimensional Fatigue Inventory (MFI-20)	3
Patient Health Questionnaire (PHQ)	3
PHQ-4	1
PHQ-9	2
Pichot Fatigue Scale (PFS)	3
Tampa Scale for Kinesiophobia	3
Chronic Pain Acceptance Questionnaire (CPAQ)	2
Chronic Pain Acceptance Questionnaire Fibromyalgia (CPAQ-FM)	1
Faces Pain Scale	2
Functional Assessment of Chronic Illness Fatigue Scale (FACIT)	2
Generalized Anxiety Disorder 7 (GAD-7)	2
International Physical Activity Questionnaire (IPAQ)	2
IPAQ	1
IPAQ-7	1
Medical Outcomes Study Sleep Scale (MOS-SLEEP)	2
Nottingham Health Profile (NHP)	2
Patient Global Impression of Improvement (PGI-I)	2
Positive Affect Negative Affect Schedule (PANAS)	2
Psychological Inflexibility in Pain Scale (PIPS)	2
Symptom Checklist 90 (SCL-90)	2
Visual Analogue Scale (VAS) Anxiety	2
Visual Analogue Scale (VAS) Depression	2
Visual Analogue Scale (VAS) Sleep	2
Visual Analogue Scale (VAS) Stiffness	2

*PROM* patient-reported outcome measure

### Reports of psychometric criteria

A proportion of 33.3% of the studies reported one or more psychometric criteria, whereas the majority (66.7%) of the studies did not present any psychometric characteristics for the collected instruments, but merely cited validation studies. In terms of reliability, the most frequently reported index for internal consistency reliability was Cronbach’s alpha. It was reported in 15.2% of the studies, although only six studies (5.7%) presented it for all instruments administered in the study [32, 60, 61, 110, 122, 124]. In four studies (3.8%) Cronbach’s alpha was calculated from the present study sample [58, 74, 97, 125]. As another aspect of reliability, test–retest reliability was reported in 8 studies (7.6%). Construct validity, including convergent and discriminant validity, was also presented in some studies. In particular, convergent validity was reported in seven (6.7%) studies [26, 74, 99, 101, 106, 122, 130]. Discriminant validity was described once [26]. Information on predictive validity was provided in one study [74]. Furthermore, sensitivity to change/responsiveness of instruments was reported in two studies [106, 123].

### Health state utilities

Instruments that allow for measuring HSU have been applied in little above half of the included studies: The SF-36 was used in 44.8% of the studies and its derivative, the SF-12 was used in 5.7% of the studies. Both can be used to calculate the preference-based SF-6D. The EQ-5D-5L was used completely in 8 studies (7.6%). Both the EQ-5D and the SF-36 were only used descriptively and HSU was not calculated in any of the studies.

## Discussion

In this systematic review we examined the selection of PROs and PROMs in (randomized) controlled trials that were published since 2015. We found that most of the studies assessed the majority of the domains that have been recommended by OMERACT. Only a minority of studies, however, followed these recommendations in full. In addition, the results show great heterogeneity in the use of PROMs across studies which reduces comparability of outcomes between these studies.

The most frequently covered domains were pain and depression. The heterogeneity and number of domains and instruments collected in FM trials were somewhat surprising. Across all included studies, 50 different PRO domains were measured by as much as 126 different instruments. Only 12 of these 50 domains were recommended by OMERACT, whereas 38 were not [5]. Whereas 18 of 50 domains were only measured once, most domains were assessed in several studies. This pronounced heterogeneity in the assessment of PROs in FM might have several reasons. First, there is still disagreement among researchers on how FM is best defined and classified [132]. Researchers who chose to use the ACR 1990 criteria, for example, might have felt that the existence of trigger points is important in FM, whereas researchers who preferred using the ACR 2010 criteria might have intended to emphasize that FM is accompanied by psychological and social problems [132]. Second, many researchers may not be aware of recommendations such as the OMERACT recommendations or do not agree with those. Third, researchers tend to use instruments that they are familiar with and that are available in their language. Fourth, the selection of the instruments and domains also clearly depends on the specific aims of the studies.

Across the investigated studies, the OMERACT recommendations were followed to varying degrees. For example, the prioritization of different layers of core domains as suggested by OMERACT does not translate to the use of domains in the studies. Most studies failed to include all mandatory domains, because tenderness was only assessed in about 17% of the studies. Remarkably, several studies preferred assessing domains that were deemed less important. In addition, a wide variety of standalone domains that were not recommended at all by OMERACT were collected.

The FIQ was by far the most frequently administered disease-specific instrument. This is not surprising because the FIQ is probably the most widely used FM instrument, it has demonstrated good psychometric properties, and it is available in several languages [8, 9, 133–136]. In addition, in its revised version, it covers many aspects of the most recent classifications. Disease-specific instruments have the advantage of focussing on aspects of a disease that are most relevant to a particular condition. Given the trend towards standardization of health outcomes, however, there are also reasons against using disease-specific instruments because the resulting scores are not easily comparable to other similar HRQOL instruments. This renders comparisons across different (similar) diseases—such as across pain conditions—inconvenient [137]. In addition, using generic health assessments may help clinicians integrate the effects of multiple conditions [138]. Maybe the most prominent example are the instruments provided by the PROMIS initiative, which allow flexible and precise assessment. The results of the present review demonstrate that the majority of domains that were assessed in the included studies were covered by the domains of the PROMIS Profile, an increasingly widely used generic health instrument.

One of the questions that motivated this study was whether instruments were used that would allow secondary cost-effectiveness analyses. These analyses are required if healthcare systems are re-modelled towards value-based healthcare [139]. We found that while about 45% of our studies used the SF-36, an instrument that can be used to calculate the preference-based HSU score of the SF-6D, only 7.6% used the EQ-5D, which is most widely used for cost-effectiveness analyses [17]. The EQ-5D measures coarsely, particularly in individuals, which is probably why it is not preferred in a clinical context [140]. The PROMIS Preference Score (PROPr), which is based on the PROMIS framework, may be an alternative for HSU measurement. In contrast to the EQ-5D and SF-6D, it covers FM-relevant domains such as fatigue and cognition [140]. Another direction could be mapping of available FM instruments to the EQ-5D or other preference-based measures. A recent paper from a spanish working group, for example, suggested that the FIQ-R might be used for calculating QALYs in FM [141]. If the mapping algorithm developed in this paper proofs reliable in future studies this would greatly increase the number of FM studies that could be used for cost-effectiveness analyses.

### Implications for future research

Our findings suggest that the current recommendations are probably not well disseminated and accepted among all FM researchers. Thus, a revised recommendation for standard outcome assessment in FM is desirable. We think that such a recommendation should include state-of-the-art PRO assessment, oriented towards standardized outcome assessment. The heterogeneous use of PROs brings several challenges to FM research. For example, it is difficult to compare treatment options from different settings if the measurement of the corresponding outcome was performed with different instruments [3, 4]. This complicates synthesizing data for meta-analyses and finally prevents the generation of treatment guidelines for FM based on these data. Clinical presentation of FM is variable and subjective. Consequently, a precise and consistent measurement of PROs is of great importance. Encouragingly, this can be achieved with existing measurement instruments (see OMERACT module update [6]).

Furthermore, we suggest that PROMs used meet certain quality criteria that reflect sufficient psychometric soundness. OMERACT, for example, provides a guideline on instrument selection, which even includes an evaluation by their technical advisory group (https://omeract.org/instrument-selection/). PROM databases might be helpful for identifying instruments and receive a quick overview of psychometric characteristics. One example is the Patient-Reported Outcome and Quality of Life Instrument Database (PROQOLID, https://eprovide.mapi-trust.org/catalog) that includes detailed information on over 4000 instruments. In any case, it is desirable that basic psychometric data on PROMs used are stated publications of FM trials and that relevant literature is carefully cited. The American Psychological Association, for example, recommends that authors provide information in the methods section of a publication on measurement instruments. This information should include the psychometric properties of the instrument to enable readers to appropriately interpret and evaluate the results of a study. Thus, the internal consistency reliability and test–retest reliability should be the minimum that is included in each clinical trial that utilizes PROMs.

### Limitations

A limitation of the present study is that only publications from 2015 to June 2022 were included and that observational studies were excluded. Furthermore, only publications in English and German language were obtained, which poses a risk of language bias. Although all decisions were made in consensus of the group, allocation and summarization of domains are to a certain extent a matter of opinion. Quality assessment usually facilitates high scientific quality of systematic reviews, especially if outcomes of studies are compared and synthesized. In this study, however, we did only investigate which PROs and PROMs were selected by researchers that were conducting clinical trials, regardless of the scientific quality of the studies. Thus, we did not perform a risk of bias analysis of the selected studies. In addition, there is some evidence that reporting of PRO data is not fully presented in articles, although the information is available and has been published in registry protocols. Therefore, measured PROs and PROMs might have been missed in this systematic review. Accordingly, simultaneous consideration of registration protocols may be a valuable addition for similar studies in the future [142]. Finally, we did not conduct a systematic literature research to identify recommendations on outcome assessment in FM but used available information from the scientific community and did ad-hoc online searches.

## Conclusion

A wide variety of PROs and PROMs were used in FM trials. A continuing attempt to standardize outcome measurement in FM is highly desirable.

## Supplementary Information

Below is the link to the electronic supplementary material.

Supplementary file1 (DOCX 70 kb)

## Data Availability

All data are incorporated into the article. Further information on conducted analyses is available on request.
