# Supplementary material for: Are OMERACT recommendations followed in clinical trials on fibromyalgia? A systematic review of patient-reported outcomes and their measures
Source: Qual Life Res. 2022 Oct 1;32(6):1521–36. doi: 10.1007/s11136-022-03261-5 (PMC10172242; doi:10.1007/s11136-022-03261-5)
Supplement: Supplementary file 1 — Supplementary file1 (DOCX 70 kb) [file 11136_2022_3261_MOESM1_ESM.docx]

**Online Supplemental Material:** Summary of characteristics of articles included in this systematic review.

| **Authors** | **Title** | **Study type** | **Trial-number** | **Country** | **Number of participants** | **Age: mean (standard deviation)**  **(in years)** | **Female**  **participants**  **in percent** | **Instruments used to assess HRQoL** |
| --- | --- | --- | --- | --- | --- | --- | --- | --- |
| Altas *et al.* (2019) [24] | Is high-frequency repetitive transcranial magnetic stimulation of the left primary motor cortex superior to the stimulation of the left dorsolateral prefrontal cortex in fibromyalgia syndrome? | RCT | not specified | Turkey | 30 | not specified | 100 | VAS (pain)  FIQ 1991  FSS  SF36  BDI |
| Altinbilek *et al*. (2019) [25] | Evaluation of the effects of neural therapy in patients diagnosed with fibromyalgia | RCT | not specified | Turkey | 72 | 39.2 (9.5) | 83.3 | VAS (pain)  FIQ 1991  BDI  BAI  SF-36 |
| Amirova *et al.* (2017) [26] | The effectiveness of the Mitchell Method Relaxation Technique for the treatment of fibromyalgia symptoms: A three-arm randomized controlled trial | RCT | not specified | England | 191 | Relaxation intervention: 48.1 (11.08); Active control: 50.46 (10.75); Usual care: 48.95 (10.13) | 93.7 | VAS (pain)  MOS-SS  SF-36 (fatigue subscale)  FIQ-R6  HADS  PSS  Two self-designed questions on coping  Treatment credibility scale |
| Andrade *et al.* (2019) [27] | Resistance Training Improves Quality of Life and Associated Factors in Patients With Fibromyalgia Syndrome | Clinical Trial | Brazilian Clinical Trials Register No. RBR-74pcmw | Brazil | 46 | 51.4 (9.34) | 100 | FIQ  PSQI short form  BDI  BAI |
| Andrade *et al.* (2017) [28] | What Is the Effect of Strength Training on Pain and Sleep in Patients With Fibromyalgia? | Clinical Trial | not specified | Brazil | 52 | 53.88 (7.68) | 96.2 | FIQ 1991  PSQI short form |
| Andrade *et al.* (2020) [29] | Preferred exercise and mental health of the patients with fibromyalgia syndrome | Clinical Trial | not specified | Brazil | 36 | Exercise intervention: 55 (7); Control: 51 (9) | Exercise  Intervention:  90.9; Control: 92.9 | FIQ  BDI  Brunel Mood Scale |
| Andrade *et al.* (2019) [30] | Effects of aquatic training and detraining on women with fibromyalgia: controlled randomized clinical trial | RCT | clinicaltrials.gov NCT01839305 | Brazil | 54 | Exercise Intervention: 48 (8); Control: 47 (8) | 100 | VAS (pain & fatigue, well-being)  FIQ  BDI  BAI  SF-36  PSQI |
| Arakaki *et al.* (2021) [31] | Strengthening exercises using swiss ball improve pain, health status, quality of life and muscle strength in patients with fibromyalgia: a randomized controlled trial | RCT | clinicaltrials.gov NCT02063750 | Brasilien | 60 | Ball training group: 47.4 (9); Stretching group: 47.3 (8.7) | 100 | VAS (pain)  FIQ-R  SF-36 |
| Aravena *et al.* (2020) [32] | Hypnotic intervention in people with fibromyalgia: A randomized controlled trial | RCT | not specified | Chile | 97 | Hypnotic intervention: 45.79 (12.11); Control 45.33 (10.79) | 96.9 | Satisfaction with Life Scale (Diener)  BPI short form  BFI  CFESDS |
| Assumpção *et al.* (2018) [33] | Muscle stretching exercises and resistance training in fibromyalgia: which is better? A three-arm randomized controlled trial | RCT | clinicaltrials.gov NCT01029041 | Brazil | 44 | Stretching intervention: 47.9 (5.3); Resistance training: 45.7 (7.7); Control: 46.9 (6.5) | 100 | VAS (pain)  FIQ (1991, Bennett 2005) SF-36 |
| Atan *et al.* (2020) [34] | Effectiveness of High-Intensity Interval Training vs Moderate-Intensity Continuous Training in Patients With Fibromyalgia: A Pilot Randomized Controlled Trial | RCT | clinicaltrials.gov NCT03924960 | Turkey | 55 | High-Intensity interval exercise: 46.57 (9.41); Moderate-intensity continuous exercise: 47.36 (8.01); Control: 52.70 (8.96) | 100 | FIQ  VAS (pain)  SF-36 |
| Barmaki *et al.* (2019) [35] | Multicenter, prospective, controlled double-blind study comparing FIB-19-01, a phytotherapy treatment for fibromyalgia, to a dietary supplement and to conventional treatment in patients suffering from fibromyalgia | Clinical trial | not specified | France | 100 | 48.3 (9.0) | 100 | FIQ (1991)  Pichot fatigue scale  PSQI  SF-12  HADS |
| Barrenengoa-Cuadra *et al.* (2021) [36] | Effectiveness of a structured group intervention based on pain neuroscience education for patients with fibromyalgia in primary care: A multicentre randomized open-label controlled trial | RCT | clinicaltrials.gov NCT03947502 | Spain | 139 | Structured group intervention: 52.3 (9.2); Control: 51.4 (10.2) | Structured Group  Intervention: 97.1;  Control: 91.3 | FIQ (1991)  BPI short form  HAQ  HADS  PCS  WPI  FM Symptom Severity Scale |
| Bilir *et al.* (2020) [37] | Effects of high frequency neuronavigated repetitive transcranial magnetic stimulation in fibromyalgia syndrome: A double-blinded, randomized controlled study | RCT | clinicaltrials.gov NCT03909009 | Turkey | 20 | 45.25 (9.08) | 100 | VAS (pain, stiffness)  FIQ (1991, Bennet 2005)  FSS  HADS |
| Böhm *et al.* (2021) [38] | High-dose spironolactone lacks effectiveness in treatment of fibromyalgia (RCT) | RCT | Eudra-CT (2014-003350-13), german clinical trial registry DRKS00008024 | Germany | 43 | Spironolactone treatment: 48.6 (13.0), Placebo Control: 46.6 (10.3) | 100 | FIQ  NRS (pain)  CES-D9  SF-36 |
| Bourgault *et al.* (2015) [39] | Multicomponent interdisciplinary group intervention for self-management of fibromyalgia: a mixed-methods randomized controlled trial | RCT | International Standard Randomized Controlled Trial Register ISRCTN14526380 | Canada | 43 | Group intervention sessions: 49.98 (9.23); Waiting list Control Group: 46.74 (11.42) | 92.6 | NRS (pain intensity)  FIQ (1991)  BPI modified  CPSI  CSQ  BDI  PCS  SF-12 Version 2  PGIC  Pain relief Scale |
| Britto *et al.* (2020) [40] | Effects of water- and land-based exercises on quality of life and physical aspects in women with fibromyalgia: A randomized clinical trial | RCT | not specified | Brazil | 33 | Water-based exercise: 50.25 (6.09); Land-based exercise: 46.18 (10.84) | 100 | FIQ (1991)  VAS (pain) |
| Calandre *et al.* (2021) [41] | The Probiotic VSL#3® Does Not Seem to Be Efficacious for the Treatment of Gastrointestinal Symptomatology of Patients with Fibromyalgia: A Randomized, Double-Blind, Placebo-Controlled Clinical Trial | RCT | clinicaltrials.gov NCT04256785 | Spain | 110 | Probiotic treatment intervention: 56.0 (7.5); Placebo Group: 55.5 (8.6) | Probiotic  Treatment  Intervention:  96.3;  Placebo Group:  98.2 | VAS (abdominal pain, abdominal bloating, meteorism, flatulence, constipation, diarrhoea, nausea, eructation, dyspepsia)  FIQ-R  ISI  PHQ-9  PGI-C  SF-36 |
| Cao *et al.* (2020) [42] | Partially randomized patient preference trial: Comparative evaluation of fibromyalgia between acupuncture and cupping therapy (PRPP-FACT) | Partially Randomized Patient Preference (PRPP) trial | clinicaltrials.gov NCT01869712 | China | 126 | Acupuncture group, randomized: 54.50; Cupping therapy group, randomized 53.00; Acupuncture group, non-randomized 55.00; Cupping therapy group, non-randomized: 53.00 | 82 | VAS (pain intensity)  HAM-D  SF-36  FIQ-R  WPI  FM Symptom Severity Scale  Two self designed items (patient satisfaction, patient expectation) |
| Castaño *et al.* (2019) [43] | Melatonin Improves Mood Status and Quality of Life and Decreases Cortisol Levels in Fibromyalgia | Clinical Trial | not specified | Spain | 33 | not exactly specified; patients had to be 40 to 60 years old to be included | 100 | FIQ (1991)  NPS (pain)  STAI  VAS (general mood status, family relationships, social relations, frame of mind, fitness)  SF-36 |
| Castel *et al.* (2015) [44] | Body mass index and response to a multidisciplinary treatment of fibromyalgia | RCT | not specified | Spain | 130 | 49.2 (6.8) | 100 | NPS (pain intensity)  HADS  CSQ (subscale catastrophizing) FIQ (1991)  MOS-SS  Dartmouth COOP functional health assessement charts (WONCA) |
| Castro Sánchez *et al.* (2019) [45] | Improvement in clinical outcomes after dry needling versus myofascial release on pain pressure thresholds, quality of life, fatigue, pain intensity, quality of sleep, anxiety, and depression in patients with fibromyalgia syndrome | RCT | clinicaltrials.gov NCT03015662 | Spain | 64 | Dry needling group: 47.37 (4.98); Myofascial release group: 46.79 (7.23) | 90.6 | SF-36  FIQ  PSQI  VAS (pain intensity, degree of relief)  STAI  BDI  FIS  HADS |
| Ceballos-Laita *et al.* (2020) [46] | Therapeutic Exercise and Pain Neurophysiology Education in Female Patients with Fibromyalgia Syndrome: A Feasibility Study | Clinical Trial | clinicaltrials.gov NCT03641495 | Spain | 32 | Exercise and educational intervention: 52.13 (10.31); Exercise intervention: 53.00 (10.68) | 100 | VAS (pain intensity) FIQ-R  PCS  HADS  HAQ-9 |
| Ceca *et al.* (2017) [47] | Benefits of a self-myofascial release program on health-related quality of life in people with fibromyalgia: a randomized controlled trial | RCT | not specified | Spain | 43 | not specified | 90.7 | FIQ (1991) |
| Chaves *et al.* (2020) [48] | Ingestion of a THC-Rich Cannabis Oil in People with Fibromyalgia: A Randomized, Double-Blind, Placebo-Controlled Clinical Trial | RCT | not specified | Brazil | 17 | 51.9 | 100 | FIQ (1991) |
| Collado-Mateo *et al.* (2017) [49] | Effects of Exergames on Quality of Life, Pain, and Disease Effect in Women With Fibromyalgia: A Randomized Controlled Trial | RCT | Australian New Zealand Clinical Trials Registry ACTRN12615000836538 | Spain | 83 | Exercise intervention: 52.52 (9.73); Control: 52.47 (8.75) | 100 | FIQ (2005 Bennet)  EQ5D-5L |
| Coste *et al.* (2021) [50] | Osteopathic medicine for fibromyalgia: a sham-controlled randomized clinical trial | RCT | clinicaltrials.gov NCT02343237 | France | 52 | Osteopathic intervention group: 51.0 (10.3); Control intervention group: 50.2 (13.9) | 94 | VAS (pain intensity)  HADS  FABQ  PCS  MFI-20  FIQ (1991)  SF-36  PGIC |
| Curatolo *et al.* (2017) [51] | Motor cortex tRNS improves pain, affective and cognitive impairment in patients with fibromyalgia: preliminary results of a randomized sham-controlled trial | RCT | not specified | Italy | 20 | 42.8 (9.87) | 100 | VAS (pain intensity)  FIQ (1991)  HADS |
| da Silva *et al.* (2018) [52] | Randomized, blinded, controlled trial on effectiveness of photobiomodulation therapy and exercise training in the fibromyalgia treatment | RCT | clinicaltrials.gov (No. not specified) | Brazil | 160 | Acute phototherapy intervention: 35 (3); Long-term phototherapy intervention: 40 (2) | 100 | VAS (pain)  FIQ  SF-36 |
| de Medeiros *et al.* (2020) [53] | Mat Pilates is as effective as aquatic aerobic exercise in treating women with fibromyalgia: a clinical, randomized and blind trial | RCT | clinicaltrials.gov NCT03149198 | Brazil | 42 | Aquatic aerobic exercise group: 50.7 (9.7); Mat pilates group: 45.5 (10.6) | 100 | VAS (pain intensity)  FIQ  PSQI  SF-36  FABQ-BR  PRCTS |
| Dias *et al.* (2016) [54] | Short-term complementary and alternative medicine on quality of life in women with fibromyalgia | Clinical Trial | not specified | Brazil | 30 | 46.90 (9.24) | 100 | WBPFS (pain intensity)  SF-36 |
| Dogru *et al.* (2019) [55] | Effects of Vitamin D Therapy on Quality of Life in Patients with Fibromyalgia | Clinical Trial | not specified | Turkey | 135 | Nutritional intervention, FM group: 38.7 (5.2); Nutritional intervention, control group: 38.03 (4.8) | 100 | FIQ-R  ASEX  BDI  VAS (pain)  SF-36 |
| Ducamp *et al.* (2022) [56] | Therapeutic Patient Education for Fibromyalgia during Spa Therapy: The FiETT Randomized Controlled Trial | RCT | clinicaltrials.gov NCT02406313 | France | 157 | Spa therapy group: 25-40y 6%, 40-60y 77%, 60-65y 17%; Spa therapy and Therapeutic Patient Education group: 25-40y 10%, 40-60y 68%, 60-65y 24% | Spa therapy  Group: 94;  Spa therapy and  Therapeutic  Patient  Education: 96 | FIQ (1991)  VAS (pain intensity relief)  Tampa Scale of kinesiophobia  PCS  WOMAC-Scale (functional limitation due to arthritis)  Pichot fatigue scale  Epworth sleepiness scale  HADS |
| Duruturk *et al.* (2015) [57] | Is balance exercise training as effective as aerobic exercise training in fibromyalgia syndrome? | RCT | not specified | Turkey | 26 | Aerobic exercise group: 48.1 (7.4); Balance exercise group: 54.0 (6.6) | 100 | VAS (pain)  FIQ |
| Efrati *et al.* (2015) [58] | Hyperbaric oxygen therapy can diminish fibromyalgia syndrome - Prospective clinical trial | Clinical Trial | clinicaltrials.gov NCT01827683 | Israel | 50 | Hyperbaric oxygen intervention group: 50.4 (10.9); Crossover group: 48.1 (11.1) | 100 | FIQ  SCL-90  SF-36 |
| Ekici *et al.* (2017) [59] | Effects of active/passive interventions on pain, anxiety, and quality of life in women with fibromyalgia: Randomized controlled pilot trial | RCT | clinicaltrials.gov (Number not specified) | Turkey | 22 | Exercise intervention group: 37.13 (6.37); Massage intervention group: 36.86 (7.73) | 100 | VAS (pain intensity) STAI  FIQ (1991 and Bennet 2005)  NPH |
| Espí-López *et al.* (2016) [60] | Effect of low-impact aerobic exercise combined with music therapy on patients with fibromyalgia. A pilot study | RCT | clinicaltrials.gov NCT02516761 | Spain | 35 | 55 (8.1) | 92.3 | Pain faces scale  BDI  FIQ (1991) |
| Espí-López *et al.* (2019) [61] | Effect of Kinesio taping on clinical symptoms in people with fibromyalgia: A randomized clinical trial | RCT | clinicaltrials.gov NCT02763332 | Spain | 35 | 52.3 (7.67) | 100 | VAS (pain intensity and comfort in different body regions)  FIQ (1991) |
| Fagerlund *et al.* (2015) [62] | Transcranial direct current stimulation as a treatment for patients with fibromyalgia: a randomized controlled trial | RCT | clinicaltrials.gov NCT01598181 | Norway | 48 | Non-invasive electrical brain-stimulation group: 49.04 (8.63); Sham control group: 48.17 (10.56) | 93.8 | FIQ (Bennett 2005)  HADS  SCL-90R  SF-36 Version 2  Four self-designed items (pain) |
| Fernandes *et al.* (2016) [63] | Swimming Improves Pain and Functional Capacity of Patients With Fibromyalgia: A Randomized Controlled Trial | RCT | clinicaltrials.gov NCT01547195 | Brazil | 75 | Swimming group: 48.3 (8.9); Walking group: 49.3 (9.2) | 100 | VAS (pain intensity)  FIQ  SF-36 |
| Forogh *et al.* (2021) [64] | Repetitive transcranial magnetic stimulation (rTMS) versus transcranial direct current stimulation (tDCS) in the management of patients with fibromyalgia: A randomized controlled trial | RCT | not specified | Iran | 30 | 45.9 (10.52) | 100 | VAS (pain)  FIQ-R  DASS-21 |
| Garrido-Ardila *et al.* (2021) [65] | Effects of Physiotherapy vs. Acupuncture in Quality of Life, Pain, Stiffness, Difficulty to Work and Depression of Women with Fibromyalgia: A Randomized Controlled Trial | RCT | clinicaltrials.gov NCT03638518 | Spain | 103 | 55.55 (8.12) | 100 | FIQ (1991)  VAS (pain, joint stiffness, difficulty to work, depression) |
| Gavilán-Carrera *et al.* (2019) [66] | Substituting Sedentary Time With Physical Activity in Fibromyalgia and the Association With Quality of Life and Impact of the Disease: The al-Ándalus Project | Clinical Trial | not specified | Spain | 407 | 51.4 (7.6) | 100 | FIQ-R  SF-36 |
| Germano Maciel *et al.* (2018) [67] | Low-level laser therapy combined to functional exercise on treatment of fibromyalgia: a double-blind randomized clinical trial | RCT | clinicaltrials.gov (Number not specified) | Brazil | 22 | Exercise and phototherapy group: 39.73 (5.25); Exercise and placebo phototherapy group: 40.36 (7.24) | 100 | VAS (pain intensity)  FIQ  BDI  WPI |
| Gómez-Hernández *et al.* (2020) [68] | Benefits of adding stretching to a moderate-intensity aerobic exercise programme in women with fibromyalgia: a randomized controlled trial | RCT | clinicaltrials.gov NCT02876965 | Spain | 64 | 54.27 (6.94) | 100 | PSQI  Epworth Sleepiness Scale  FIQ (1991)  VAS (pain intensity) |
| Guggino *et al.* (2020) [69] | T helper 1 response is correlated with widespread pain, fatigue, sleeping disorders and the quality of life in patients with fibromyalgia and is modulated by hyperbaric oxygen therapy | Clinical Trial | not specified | Italy | 56 | Hyperbaric oxygen intervention, FM group: 36 (8); FM Control (no treatment): 38 (10); non-FM control (no treatment): 37 (7); Hyperbaric oxygen intervention, non-FM group: 39 (4) | Hyperbaric oxygen  intervention,  FM group: 90.9;  FM Control  (no treatment): 100;  non-FM Control  (no treatment): 90;  Hyperbaric oxygen  intervention,  non-FM group: 80 | VAS (pain intensity, fatigue)  FACIT Scale  HAQ  PSQI  WPI  FM Symptom Severity Scale |
| Guinot *et al.* (2019) [70] | Effects of repetitive transcranial magnetic stimulation and multicomponent therapy in patients with fibromyalgia: a randomized controlled trial | RCT | clinicaltrials.gov NCT01308801 | France | 36 | Active non-invasive brain stimulation group: 46.5 (10.4); Sham non-invasive brain stimulation group: 42.8 (8.8) | Active non-invasive  brain stimulation  group: 100;  Sham non-invasive  brain stimulation  group: 79 | VAS (pain)  FIQ (1991)  BDI  PSQI  PCS  PGIC |
| Gulsen *et al.* (2020) [71] | Effect of fully immersive virtual reality treatment combined with exercise in fibromyalgia patients: a randomized controlled trial | RCT | not specified | Turkey | 16 | Exercise and virtual reality treatment intervention group: 38.50; Exercise intervention group: 46.50 | 100 | VAS (pain)  FIQ 1991  FSS  IPAQ  SF-36  Tampa Scale of Kinesiophobia |
| Hadanny *et al.* (2018) [72] | Hyperbaric oxygen therapy can induce neuroplasticity and significant clinical improvement in patients suffering from fibromyalgia with a history of childhood sexual abuse—randomized controlled trial | RCT | clinicaltrials.gov NCT03376269 | Israel | 30 | 45.9 (10.8) | 100 | FIQ (1991)  SF-36  BSI-18  CTQ  WPI  FM Symptom Severity Scale |
| Haugmark *et al.* (2021) [73] | Effects of a mindfulness-based and acceptance-based group programme followed by physical activity for patients with fibromyalgia: a randomised controlled trial | RCT | BMC Registry ISRCTN96836577 | Norway | 170 | 42 (24.52) | 94.0 | PGIC  NRS (pain, fatigue, very bad sleep)  GHQ-12  FFMQ  Exercise Beliefs and Exercise Habits Questionnaire  WPAI:GH  EQ-5D-5L |
| Hedman-Lagerlöf *et al.* (2018) [74] | Internet-Delivered Exposure Therapy for Fibromyalgia: A Randomized Controlled Trial | RCT | clinicaltrials.gov NCT02638636 | Sweden | 140 | 50.3 (10.9) | 98 | FIQ 1991 (Bennett 2005)  FSS  WHO-DAS  BBQ  PHQ-9  GAD-7  ISI  PRS  FFMQ-NR (subscale nonreactivity to inner experiences)  PIPS  PGIC |
| Ibáñez-Vera *et al.* (2020) [75] | Effects of Monopolar Dielectric Radiofrequency Signals on the Symptoms of Fibromyalgia: A Single-Blind Randomized Controlled Trial | RCT | Australian New Zealand Clinical Trial Registry ACTRN126170001499370 | Spain | 66 | 47 (17.7) | 100 | VAS (pain)  HADS  ICAF |
| Izquierdo-Alventosa *et al.* (2021) [76]; (2020) [77] ^a^ | Effectiveness of High-Frequency Transcranial Magnetic Stimulation and Physical Exercise in Women With Fibromyalgia: A Randomized Controlled Trial | RCT | clinicaltrials.gov NCT03801109 | Spain | 49 | 52.84 (8.31) | 100 | VAS (pain)  Borg Scale (effort during intervention)  FIQ (1991)  WPI  FM Symptom Severity Scale  PCS  HADS (anxiety scale used)  BDI-II  PSS-10  Physical function subscale of the FIQ-R  CPAQ |
|  | Low-Intensity Physical Exercise Improves Pain Catastrophizing and Other Psychological and Physical Aspects in Women with Fibromyalgia: A Randomized Controlled Trial | RCT | clinicaltrials.gov NCT03801109 | Spain | 32 | Exercise intervention: 53.06 (8.4); No intervention control group 55.13 (7.35) | 100 |  |
| Kaplun *et al.* (2021) [78] | Effects of Brief Guided Imagery on Female Patients Diagnosed with Fibromyalgia: An Exploratory Controlled Trial | Clinical Trial | NIH: Unique Protocol ID: 0015-16COM2 | Israel | 13 | 58.7 (8.8.) | 100 | BPI  SF-36 |
| Larsson *et al.* (2015) [79] | Resistance exercise improves muscle strength, health status and pain intensity in fibromyalgia-a randomized controlled trial | RCT | clinicaltrials.gov NCT01226784 | Sweden | 130 | Exercise intervention group: 50.81 (9.05); Active control group (relaxation therapy): 52.10 (9.78) | 100 | FIQ 1991 (Bennet 2005)  VAS (pain intensity)  SF-36  PDI  CPAQ  FABQ  PGIC |
| Lauche *et al*. (2016) [80] | Efficacy of cupping therapy in patients with the fibromyalgia syndrome-a randomised placebo controlled trial | RCT | clinicaltrials.gov NCT01635634 | Germany | 141 | 55.8 (9.1) | 98.6 | VAS (patient expectation of improvement)  VAS (pain intensity)  FIQ (1991)  SF-36  MFI-20  PSQI |
| Leombruni *et al.* (2015) [81] | A randomised controlled trial comparing duloxetine and acetyl L-carnitine in fibromyalgic patients: preliminary data | RCT | not specified | Italy | 51 | 51.78 (10.17) | 100 | MADRS  HADS  VAS (pain intensity)  Distress Thermometer  SF-36 |
| Lumley *et al.* (2017) [82] | Emotional awareness and expression therapy, cognitive behavioral therapy, and education for fibromyalgia: a cluster-randomized controlled trial | RCT | clinicaltrials.gov NCT01287481 | USA | 230 | 49.13 (12.22) | 93.9 | BPI  PSQI  MASQ  CESD  GAD-7  PROMIS Fatigue short form  SF-12 physical component score  Positive Affect Negative Affect Schedule  Satisfaction with Life Scale  PGIC  WPI  FM Symptom Severity Scale |
| Macian *et al.* (2022) [83] | Short-Term Magnesium Therapy Alleviates Moderate Stress in Patients with Fibromyalgia: A Randomized Double-Blind Clinical Trial | RCT | clinicaltrials.gov NCT03887000 | France | 76 | Magnesium intervention group: 54.0 (11.5); Placebo control group: 51.8 (10.9) | not specified | DASS-42 Scale  NRS (pain)  BPI  PSQI  SF-12  FSS  PCS  EPICES (social vulnerability) |
| Maddali Bongi *et al.* (2016) [84] | Efficacy of rehabilitation with Tai Ji Quan in an Italian cohort of patients with Fibromyalgia Syndrome | Clinical Trial | not specified | Italy | 50 | 52.24 (12.19) | not specified | FIQ (1991)  SF-36  HAQ  FACIT-fatigue  PSQI  HADS  WPI |
| Mahagna *et al.* (2016) [85] | A randomised, double-blinded study comparing giving etoricoxib vs. placebo to female patients with fibromyalgia | RCT | clinicaltrials.gov NCT00755521 | Israel | 64 | Drug intervention group: 49.8 (13.2);  Placebo control group: 51.0  (9.7) | 100 | SF-36  BPI  FIQ (1991)  HAM-A  HAM-D |
| Maindet *et al.* (2021) [86] | Spa Therapy for the Treatment of Fibromyalgia: An Open, Randomized Multicenter Trial | RCT | clinicaltrials.gov NCT02265029 | France | 218 | Spa therapy group: 50.4 (8.9), Control group: 49.2 (8.8) | Spa therapy group:  90; Control group:  91.7 | FIQ (1991)  EQ-5D-3L  PGA  VAS (pain)  PCS  PSQI  Epworth Sleepiness Scale  Pichot Fatigue Scale  HADS  Coping Questionnaire  Baecke Questionnaire (physical activity)  WPI  FM Symptom Severity Scale |
| Mantia *et al*. (2015) [87] | Shortness of filum terminale represents an anatomical specific feature in fibromyalgia: a nuclear magnetic resonance and clinical study | Clinical Trial | not specified | Italy | 38 | Surgery and physical therapy group: 51 (6); Physical therapy group: 52 (5.1) | 94.7 | FIQ (1991)  FAS |
| Matias *et al.* (2022) [88] | Transcranial Direct Current Stimulation Associated With Functional Exercise Program for Treating Fibromyalgia: A Randomized Controlled Trial | RCT | registered on ensaclinicos.gov.br | Brazil | 31 | Transcranial direct current stimulation group: 48.9 (13.8); Sham intervention group: 49.4 (15.1) | 100 | VAS (pain, anxiety)  FIQ (1991)  BDI  MFI-20  Feeling Scale (affective valence) |
| Mendonca *et al.* (2016) [89] | Transcranial Direct Current Stimulation Combined with Aerobic Exercise to Optimize Analgesic Responses in Fibromyalgia: A Randomized Placebo-Controlled Clinical Trial | RCT | clinicaltrials.gov NTC02358902 | Brazil | 45 | 47.4 (12.1) | 97.8 | VNS (pain intensity, anxiety)  SF-36  BDI |
| Miki *et al.* (2016) [90] | Efficacy of mirtazapine for the treatment of fibromyalgia without concomitant depression: a randomized, double-blind, placebo-controlled phase IIa study in Japan | RCT | clinicaltrials.jp JapicCTI-122,005 | Japan | 422 | Mirtazapine treatment intervention group: 45.0 (10.4); Placebo Control: 45.3 (10.3) | Mirtazapine  treatment  intervention group:  82.5;  Placebo control  group: 82.0 | NRS (pain)  FIQ  SF-36  BDI-II  ISI  PSQI |
| Mingorance *et al.* (2021) [91]  Mingorance *et al.* (2021) [92] ^a^ | A Comparison of the Effect of Two Types of Whole Body Vibration Platforms on Fibromyalgia. A Randomized Controlled Trial | RCT | clinicaltrials.gov NCT03782181 | Spain | 60 | 52.4 (8.4) | 90 | FIQ (1991)  VAS (pain)  Quality of Life Index |
|  | The Therapeutic Effects of  Whole-Body Vibration in Patients  With Fibromyalgia. A Randomized  Controlled Trial | RCT | clinicaltrials.gov NCT03782181 | Spain | 40 | 52.5 (8.3) | 90 |  |
| Mirzaei *et al.* (2018) [93] | Effects of vitamin D optimization on quality of life of patients with fibromyalgia: A randomized controlled trial | RCT | Iranian Registry of Clinical Trials IRCT2012100610960N2 | Iran | 74 | 41.6 (10.5) | 100 | SF- 36  PSQI  FIQ  WPI |
| Montero-Marín *et al.* (2018) [94] | Efficacy of 'attachment-based compassion therapy' in the treatment of fibromyalgia: A randomized controlled trial | RCT | clinicaltrials.gov NCT02454244 | Spain | 42 | Therapeutic intervention group: 50.83 (8.70); Active control group (relaxation therapy): 52.21 (5.95) | 100 | FIQ  PCS  HADS  VAS of the EQ-5D  AAQ-II |
| Montesó-Curto *et al.* (2015) [95] | Effectiveness of Three Types of Interventions in Patients with Fibromyalgia in a Region of Southern Catalonia | RCT | not specified | Spain | 66 | 58.9 (10.9) | 96.9 | EQ-5D  SCOPA  Plutchik suicide risk scale  VAS (pain intensity)  Satisfaction with the Infiltration Scale |
| Murakami *et al.* (2015) [96] | A randomized, double-blind, placebo-controlled phase III trial of duloxetine in Japanese fibromyalgia patients | RCT | clinicaltrials.gov NCT01552057 | Japan | 386 | Duloxetine treatment intervention group: 47.8 (12.0); Placebo control group: 49.5 (11.7) | Duloxetine  treatment  intervention group:  82.2; Placebo  control group:  84.1 | BPI  PGIC-I  FIQ (1991)  SF-36  WPI  FM Symptom Severity Scale  BDI-II |
| Musekamp *et al.* (2019) [97] | Evaluation of a self-management patient education programme for fibromyalgia-results of a cluster-RCT in inpatient rehabilitation | RCT | German Clinical Trials Register DRKS00008782 | Germany | 583 | Patient-education intervention group: 51.5 (7.5); Active control group (treatment as usual): 51.7 (8.6) | Patient-education  Intervention group:  93.4; Active control  Group (treatment  as usual): 95.4 | Patient Education Satisfaction  Questionnaire  heiQ  Rheuma  ControlScale  Godin Leisure-Time Exercise Questionnaire, relaxation index  FIQ  PHQ-4  FPQ |
| Nadal-Nicolás *et al.* (2020] [98] | Effects of Manual Therapy on Fatigue, Pain, and Psychological Aspects in Women with Fibromyalgia | RCT | clinicaltrials.gov NCT04158388 | Spain | 24 | 53 (6) | 100 | FSS  VAS (pain perception)  PSQI  POMS-29 |
| Onieva-Zafra *et al.* (2015) [99] | Effectiveness of guided imagery relaxation on levels of pain and depression in patients diagnosed with fibromyalgia | Clinical trial | not specified | Spain | 55 | 52.47 (6.17) | 96.4 | MPQ  VAS (pain, depression)  BDI |
| Onieva-Zafra *et al.* (2019) [100] | Benefits of a Home Treatment Program Using Guided Imagery Relaxation Based on Audio Recordings for People With Fibromyalgia | Clinical trial | not specified | Spain | 55 | 52.47 (6.17) | 96.4 | STAI  PSQI  Chronic Pain Efficacy Scale  SF-36  FIQ (1991) |
| Ozen *et al.* (2019) [101] | A Comparison of Physical Therapy Modalities Versus Acupuncture in the Treatment of Fibromyalgia Syndrome: A Pilot Study | Clinical Trial | not specified | Turkey | 44 | 48.1 (8.77) | 100 | SF-MPQ  FIQ (1991) |
| Pagliai *et al.* (2020) [102] | Effectiveness of a Khorasan Wheat-Based Replacement on Pain Symptoms and Quality of Life in Patients with Fibromyalgia | RCT | not specified | Italy | 20 | 48.9 (12.3) | 95 | FIQ (1991)  FSS  TSS  SRSBQ  RSQD  FOSQ  WPI  FM Symptom Severity Scale |
| Pătru *et al.* (2020) [103] | Influence of multidisciplinary therapeutic approach on fibromyalgia patients | RCT | not specified | Romania | 98 | Cognitive behavioral and occupational therapy group: 52.5 (7.2); Kinetic therapy group: 55.3 (7.2); Control group: 56.3 (8.8) | 100 | FIQ (1991)  Fibro Fatigue Scale |
| Park *et al*. (2021) [104] | Comparison of core muscle strengthening exercise and stretching exercise in middle-aged women with fibromyalgia: A randomized, single-blind, controlled study | RCT | Clinical Research Information Service CRS: KCT0003111 | South Korea | 40 | Strengthening intervention group: 52.8 (7.1); Stretching intervention group: 50.5 (7.1) | 95 | VAS (pain)  Borg Scale (effort during intervention)  FIQ (1991)  WPI  FM Symptom Severity Scale |
| Pazzi *et al.* (2020) [105] | Ganoderma lucidum Effects on Mood and Health-Related Quality of Life in Women with Fibromyalgia | RCT | New Zealand Clinical Trials Registry ACTRN12614001201662 | Spain | 70 | Ganoderma lucidum treatment intervention group: 56.19 (7.97); Placebo control group: 53.74 (11.50) | 95.7 | SHS  SWLS  GDS  SF-12  GIIS |
| Pérez-Aranda *et al.* (2019) [106] | A randomized controlled efficacy trial of mindfulness-based stress reduction compared with an active control group and usual care for fibromyalgia: the EUDAIMON study | RCT | clincialtrials.gov NCT02561416 | Spain | 225 | Mindfulness-based therapy intervention group: 52.96 (7.98); non-mindfulness therapy intervention group: 54.21 (7.41); Control group (no Intervention): 52.65 (8.52) | Mindfulness-based  therapy  intervention group:  97.3;  non-mindfulness-  based therapy  intervention group:  98.7; Control group  (no intervention):  98.7 | FIQ-R  WPI  FM Symptom Severity Scale  HADS  PCS  PSS-10  MISCI  FFMQ  SCS-12  PIPS  PGIC  PSIC  CEQ |
| Plazier *et al.* (2015) [107] | C2 Nerve Field Stimulation for the Treatment of Fibromyalgia: A Prospective, Double-blind, Randomized, Controlled Cross-over Study | RCT | clinicaltrials.gov NCT00917176 | Belgium | 40 | Temporary subcutaneous C2 nerve field stimulation group: 45.4 (9.8); Permanent subcutaneous C2 nerve field stimulation group: 46.4 (10.1) | Temporary  subcutaneous C2  nerve field  stimulation group:  85.7; Permanent  subcutaneous  nerve field  stimulation group:  88.0 | FIQ (Bennett 2005)  PVAQ  PCS  NRS (pain, HRQoL)  BDI  Modified Fatigue Impact Scale (MFIS)  PSQI |
| Polat *et al*. (2021) [108] | The Effect of Virtual Reality Exercises on Pain, Functionality, Cardiopulmonary Capacity, and Quality of Life in Fibromyalgia Syndrome: A Randomized Controlled Study | RCT | not specified | Turkey | 40 | Virtual reality intervention group: 42.6 (8.7); Control group: 47.0 (7.1) | 100 | FIQ (1991)  VAS (pain intensity)  HADS  Fatigue Severity Scale  FM Symptom Severity Scale  VAS Index of EQ5D-3L |
| Ramzy (2017) [109] | Comparative Efficacy of Newer Antidepressants in Combination with Pregabalin for Fibromyalgia Syndrome: A Controlled, Randomized Study | RCT | clinicaltrials.gov NCT02451475 | Egypt | 75 | Amitriptyline treatment intervention group: 56.9 (6.82); Venlafaxine treatment intervention group: 44.0 (6.30); Paroxetine treatment intervention group: 46.2 (7.60) | 100 | SSS-8  CESDS |
| Rodríguez-Mansilla *et al.* (2021) [110] | Effects of Non-Pharmacological Treatment on Pain, Flexibility, Balance and Quality of Life in Women with Fibromyalgia: A Randomised Clinical Trial | RCT | clinicaltrials.gov NCT04328142 | Spain | 93 | 52.24 (6.19) | 100 | VAS (pain)  FIQ (1991)  Borg Scale of Perceived Exertion |
| Salaffi *et al.* (2020) [111] | Exercise therapy in fibromyalgia patients: comparison of a web-based intervention with usual care | Clinical Trial | not specified | Italy | 140 | 50.63 (8.94) | 88.6 | FIQ-R  Modified FAS |
| Samartin-Veiga *et al.* (2022) [112] | Active and sham transcranial direct current stimulation (tDCS) improved quality of life in female patients with fibromyalgia | RCT | encepp.eu Code: 2014/288 | Spain | 108 | Stimulation over primary motorcortex group: 49.38 (8.83); Stimulation over dorsolateral prefrontal cortex group: 51.0 (9.15); Stimulation over operculo-insula cortex group: 50.21 (8.2); Sham intervention control group: 50.67 (8.88) | 100 | SF-36  FIQ-R  WPI  FM Symptom Severity Scale |
| Saral *et al.* (2016) [113] | The effects of long- and short-term interdisciplinary treatment approaches in women with fibromyalgia: a randomized controlled trial | RCT | not specified | Turkey | 59 | Long-term inter-disciplinary intervention group: 38.3 (9.8); short-term inter-disciplinary intervention group: 43.2 (9.2); Control group (no intervention): 43.7 (1.1) | 100 | VAS (pain intensity, fatigue, sleep quality)  FIQ (1991)  BDI  SF-36 |
| Sarmento *et al.* (2020) [114] | The therapeutic efficacy of Qigong exercise on the main symptoms of fibromyalgia: A pilot randomized clinical trial | RCT | clinicaltrials.gov NCT03441997 | USA | 20 | Qigong exercise intervention group: 42.6 (10.7); Control exercise intervention group: 56.1 (12.3) | 100 | SF-MPQ  VAS (pain)  FIQ-R  PSQI  HADS  QOLS |
| Sauch Valmaña *et al.* (2020) [115] | Effects of a Physical Exercise Program on Patients Affected with Fibromyalgia | Clinical Trial | not specified | Spain | 50 | 53.95 (7.698) | 100 | VAS (pain)  SF-36  FIQ (1991) |
| Scaturro *et al*. (2019) [116] | An intense physical rehabilitation programme determines pain relief and improves the global quality of life in patients with fibromyalgia | Clinical Trial | not specified | Italy | 60 | 49 (5.7) | 100 | FIQ (1991)  SF-36  VAS (pain, fatigue)  WPI  FM Symptom Severity Scale |
| Schweiger *et al.* (2020) [117] | Comparison between Acupuncture and Nutraceutical Treatment with Migratens(®) in Patients with Fibromyalgia Syndrome: A Prospective Randomized Clinical Trial | RCT | clinicaltrials.gov NCT04098757 | Italy | 60 | Nutritional-supplement intervention group: 48.2 (7.4); Acupuncture intervention group: 52.9 (8.5) | 100 | VAS (pain)  FIQ-R  WPI  FM Symptom Severity Scale |
| Silva *et al.* (2019) [118] | Sophrology versus resistance training for treatment of women with fibromyalgia: A randomized controlled trial | RCT | not specified | Brazil | 60 | Exercise intervention group: 44.93 (10.30); Relaxation intervention group: 49.40 (8.30) | 100 | VAS (pain)  FIQ (1991)  SF-36 |
| Suttiruksa *et al.* (2016) [119] | Effects of mirtazapine on quality of life of Thai patients with fibromyalgia syndrome: A double-blind, randomized, placebo-controlled trial | RCT | clinicaltrials.gov NCT00919295 | Thailand | 40 | Mirtazapine treatment intervention group: 43.4 (56.6); Placebo control group: 47.4 (37.9) | 100 | SF-36 |
| Tomas-Carus *et al.* (2022) [120] | Effects of respiratory muscle training on respiratory efficiency and health-related quality of life in sedentary women with fibromyalgia: a randomised controlled trial | RCT | ISRCTN37081460 | Portugal | 30 | Exercise intervention group: 54.1 (9.3); Control group: 50.8 (8.7) | 100 | SF-36 |
| Toprak Celenay *et al.* (2017) [121] | A comparison of the effects of exercises plus connective tissue massage to exercises alone in women with fibromyalgia syndrome: a randomized controlled trial | RCT | Clinical Researches Ankara, Turkey Approval number: 105/2015 | Turkey | 40 | Exercise intervention: 39.9 (9.5); Exercise and massage intervention: 42.5 (8.3) | 100 | FIQ (1991)  VAS (pain, fatigue, sleep)  IPAQ-7  SF-36 |
| Torres *et al.* (2018) [122] | Randomized Trial of a Group Music and Imagery Method (GrpMI) for Women with Fibromyalgia | Clinical Trial | not specified | Spain | 56 | 51.34 (7.37) | 100 | PWS  FIQ (1991)  MPQ  STAI  ST-DEP |
| Torres *et al*. (2015) [123] | Results of an Active Neurodynamic Mobilization Program in Patients With Fibromyalgia Syndrome: A Randomized Controlled Trial | RCT | clinicaltrials.gov NCT01826695 | Spain | 48 | Mobilization intervention group: 53.0 (10.27); Control group (no intervention): 53.10 (7.66) | Mobilization  Intervention group:  80; Control group:  82.6 | BAI  BDI  FIQ-R  BPI  PCS  HAQ-DI  Fatigue Severity Scale |
| Udina-Cortés *et al.* (2020) [124] | Effects of neuro-adaptive electrostimulation therapy on pain and disability in fibromyalgia | RCT | clinicaltrials.gov NCT03882567 | Spain | 37 | 52 (8) | 100 | VAS (pain)  FIQ (1991)  SF-36  Jenkins Sleep Scale  PCS  STAI  BDI  Tampa Scale of Kinesiophobia |
| van Eijk-Hustings *et al.* (2015) [125] | Predictors for health improvement in patients with fibromyalgia: a 2-year follow-up study | RCT | not specified | Netherlands | 203 | Multi-disciplinary intervention: participants: 42.0 (8.8), non-participants: 40.8 (10.7); Aerobic exercise: participants: 43.6 (8.7), non-participants: 40.5 (9.1); Usual care control group: 42.9 (11.0) | Multi-disciplinary  intervention:  Participants: 93.3,  non-participants:  93.8;  Aerobic exercise:  participants: 100  non-participants:  100  Usual care control  group:  97.9 | PGI-C  VAS of EQ-5D (EQ-VAS)  FIQ (2005)  General Self-Efficacy Scale CIS-20)  PCS  PANAS  PASS-20 |
| Vas *et al.* (2016) [126] | Acupuncture for fibromyalgia in primary care: a randomised controlled trial | RCT | International Standard Randomised Controlled Trial Number ISRCTN60217348 | Spain | 162 | 52.8 (9.6) | 100 | VAS (pain intensity)  SF-12  FIQ  One self-designed item (improvement perceived by the participant) |
| Vayvay *et al.* (2016) [127] | The effect of Laser and taping on pain, functional status and quality of life in patients with fibromyalgia syndrome: A placebo- randomized controlled clinical trial | RCT | not specified | Turkey | 45 | Laser intervention group: 36.4 (8.3); Taping intervention group: 38 (9.9); Placebo laser intervention control group: 38 (8.4) | 100 | VAS (pain intensity)  FIQ  SF-36  BDI |
| Villafaina *et al.* (2019) [128] | Benefits of 24-Week Exergame Intervention on Health-Related Quality of Life and Pain in Women with Fibromyalgia: A Single-Blind, Randomized Controlled Trial | RCT | International Standard Randomised Controlled Trial Number ISRCTN65034180 | Spain | 25 | Exercise intervention group: 54.04 (8.45); Control group (no intervention): 52.72 (9.98) | 100 | EQ-5D-5L  VAS (pain intensity) |
| Vitenet *et al.* (2018) [129] | Effect of whole body cryotherapy interventions on health-related quality of life in fibromyalgia patients: A randomized controlled trial | RCT | not specified | France | 24 | Cryotherapy intervention group: 55 (10); Control group (no intervention): 50 (11) | Cryotherapy  Intervention group:  72.7;  Control group (no  intervention):  92.3 | SF-36 |
| Wang *et al.* (2018) [130] | Effect of tai chi versus aerobic exercise for fibromyalgia: comparative effectiveness randomized controlled trial | RCT | clinicaltrials.gov NCT01420640 | USA | 226 | 12-week Tai chi intervention group: 53.0 (12.6), 2x12-week Tai chi intervention group: 52.1 (10.3); 24-week Tai Chi intervention group: 50.8 (11.8): 2x24-week Tai chi intervention group: 52.1 (13.3); 2x24-week Aerobic exercise intervention group: 50.9 (12.5) | 92 | FIQ-R  HADS  PSQI  arthritis self efficacy scale  SF-36  FM Symptom Severity Scale  BDI-II  Coping Strategies Questionnaire  Social Support Survey  HAQ  Improved HAQ  Outcome expectations scale  CHAMPS  Physical Activity Questionnaire |

^a^ these two records are based on the same study and therefore only included in the analysis once

AAQI-II, Acceptance and Action Questionnaire; ASEX, Arizona sexual experience scale; BAI, Beck’s Anxiety Inventory; BBQ, Brunnsviken Brief Quality of Life Scale; BDI, Beck’s Depression Inventory; BFI, Brief Fatigue Inventory; BPI, Brief Pain Inventory; BSI-18, Brief Symptom Inventory 18; CES-D, Center for Epidemiological Studies-Depression Scale; CESDS, Center for Epidemiological Studies Depression Scale; CEQ, Credibility/Expectancy Questionnaire; CGI-S, Clinical Global Impression-Severity Scale; CHAMPS, Community Health Activities Model Program for Seniors; CIS-20, Checklist Individual Strength; CPSI, Chronic Pain Sleep Inventory; CPAQ, Chronic Pain Acceptance Questionnaire; CSQ, Coping Strategy Questionnaire; CTQ,  Childhood Trauma Questionnaire; DASS-21, Depression Anxiety and Stress Scale 21; DASS-42, Depression Anxiety and Stress Scale 42; EPICES, Évaluation de la Précarité et des Inégalités de santé dans les Centres d’Examen de Sant; EQ5D-5L, European Quality of Life 5 Dimensions 5 Level; EQ5D-3L, European Quality of Life 5 Dimensions 3 Level; FABQ, Fear Avoidance Beliefs Questionnaire; FACIT, Functional Assessment of Chronic Illness Therapy; FAS, Fibromyalgia Assessment Status; FFMQ,   Five Facets of Mindfulness Questionnaire; FIQ, Fibromyalgia Impact Questionnaire; FIQ-R, Revised Fibromyalgia Impact Questionnaire; FIS, Fatigue Impact Scale; FM, Fibromyalgia; FOSQ, Functional Outcome of Sleep Questionnaire; FPQ, Fibromyalgia Participation Questionnaire; FSS, Fatigue Severity Scale; GAD-7, Generalizied Anxiety Disorder Scale; GDS, Geriatric Depression Scale; GHQ-12, General Health Questionnaire 12; GIIS, Global Impression of Improvement Scale; HADS, Hamilton Anxiety and Depression Scale; HAM-A, Hamilton Anxiety Rating Scale; HAM-D, Hamilton Depression Scale; HAQ-9, Health Assessment Questionnaire; HAQ-DI, Health Assessment Questionnaire Disability Index; HAQ-I, Improved Health Assessment Questionnaire; HRQoL, Health Related Quality of Life; ICAF, Combined Index of Fibromyalgia Severity; IPAQ, International Physical Activity Questionnaire; ISI, Insomia Severity Index; MADRS, Montgomery Asberg Depression Rating Scale; MASQ, Multiple Ability Self-Report Questionnaire; MFI-20, Multidimensional Fatigue Inventory; MFIS, Modified Fatigue Impact Scale; MISCI, Multidimensional Inventory of Subjective Cognitive Impairment; FAS, Fibromyalgia Assessment Status Questionnaire; Mod. FAS, modified Fibromyalgia Assessment Status Questionnaire; MOS-SS, Medical Outcome Study Sleep Scale; MPQ, McGill Pain Questionnaire; NPH, Nottingham Health Profile; NRS, Numeric Rating Scale; NPS, Numeric Pain Scale; PANAS, Positive Affect Negative Affect Schedule; PASS-20, Pain Anxiety Symptoms Scale; PCS, Pain Catastrophizing Scale; PDI, Pain Disability Index; PGIC, Patient Global Impression of Change Scale; PGIC-I, Patient Global Impression of Improvement; PHQ-9, Patient Health Questionnaire-9; PIPS, Psychological Inflexibility in Pain Scale; POMS-29, Profile of Mood Scales 29; PRCTS, Pain-Related Catastrophizing Thoughts Scale; PRS, Pain Reactivity Scale; PSQI, Pittsburgh Sleep Quality Inventory; PSIC, Pain-Specific Impression of Change; PSS-10, Perceived Stress Scale 10; PVAQ, Pain Vigilance and Awareness Questionnaire; PWS, Psychological Wellbeing Scale; RCT, Randomised Controlled Trial; RSQD, Restorative Sleep Questionnaire–Daily; SCOPA, Sleep Quality Questionnaire; SCS-12, Self-Compassion Scale - Short Form; SF-36, Medical Outcomes Study Short Form 36 Questionnaire; SF-12, Medical Outcomes Study Short Form 12 Questionnaire; SHS, Subjective Happiness Scale; SCL-90, Symptom severity Check List 90; SF-MPQ, Short Form McGill Pain Questionnaire; SRSBQ, Sleep-Related and Safety Behaviour Questionnaire; SSS, Somatic Symptom Scale; SSS-8, Somatic Symptom Scale-8; STAI, State-Trait Anxiety Test; ST/DEP, State-Trait Depression Questionnaire, SWLS, Satisfaction with Life Scale; THC, Δ^9^-Tetrahydrocannabinol; tRNS, transcranial random noise stimulation; TSS Tiredness Symptoms Scale; VAS, Visual Analogue Scale; VNS, Visual Numeric Scale; WBFPS, Wong-Baker Faces Pain Scale; WHO-DAS, World Health Organisation Disability Assessment Schedule; WOMAC-Scale, Osteoarthritis Index of the Western Ontario and McMaster University; WPAI:GH, Work Productivity and Activity Impairment General Health Version 2.1.; WPI, Widespread Pain Index
